# Supplementary material for: Multiple clades of Husavirus in South America revealed by next generation sequencing
Source: PLoS One. 2021 Mar 23;16(3):e0248486. doi: 10.1371/journal.pone.0248486 (PMC7987173; doi:10.1371/journal.pone.0248486)
Supplement: Attachment — Submitted filename: Review (PLOS ONE).docx [file pone.0248486.s001.docx]

**Review**

**PLOS One Journal**

**Multiple clades of Husavirus in South America revealed by next-generation sequencing of faecal samples**

The authors studied the molecular characterization of husavirus in faecal samples from patients with gastroenteritis in South America. They detected husavirus in only five samples. The authors showed that the viral strains are closely related based on their nucleotide sequence and are closely related to a HuV identified in Viet Nam in 2013. In general, the manuscript sounds good. Although there is nothing novel about the idea nor the techniques which were used to study the molecular characterization of the virus, it is a good study as it is the first on this virus in Brazil. The data of this study may add to global knowledge about the molecular epidemiology of Husavirus. There are crucial points that the authors should elucidate.

**Introduction:**

The authors should restructure the introduction as it lacks detailed information in addition to poor English.

1. How was husavirus discovered in 2015 from human faecal samples? What were the techniques used?
2. The authors stated that the virus could cause a variety of clinical conditions in the host, which host precisely? Human or non-human? Need more resources and references.
3. What is the importance of studying the phylogenetic characterization of the virus?
4. More references should be included in the introduction related to the clinical importance of the virus.

**Study population and specimen collection:**

- The statement `the protocol used failed to identify possible outbreak` needs more clarification.

**Sample screening:**

- It is recommended to screen the stool samples first for the presence of common viruses causing gastroenteritis by a routine diagnostic test such as multiplex-Real Time PCR and then perform NGS to identify the known or unknown viruses in the samples.

**Results:**

1. The authors mentioned that 251 samples were analyzed by serology or NGS; however, they did not mention that they used serology in the methodology section.
2. The authors did not remark what is the percentage of the identified HuV in the stool samples?
3. Different viruses were detected in the clinical samples using NGS. Were they found as single viruses or co-infection?
4. ‘The highest similarity of the Brazilian sequences and previously described husavirus was 87%.......’. This statement should be restructured to be more understandable.
5. It should be noted to the authors that all identified husaviruses from stool samples belong to clade A1 only, while clade A2 consists of reference husavirus A strains.
6. The authors have just included seven human husavruses to perform the phylogenetic analysis. Where are the remaining 10 human HuV nucleotides sequences that are deposited in the GeneBank database? Including all the human 17 HuV nucleotides sequences will give a better image on the relatedness of the detected strains in this study to the other strains detected in other countries.

**Discussion:**

1. The authors stated ‘the presence of a very divergent clade of HuV (clade A2) in South America, which suggest that husavirus was circulating in the native population’. This statement needs lots of explanations and confirmations. What are the other molecular analysis data that support this conclusion?
2. Since the HuV strains detected in this study were found as co-infection with other viruses causing gastroenteritis, how can the authors confirm that HuV able to cause clinical outcome as a sole virus? It may be dependant virus!
3. The authors did not discuss the limitations of this study.
