# Supplementary material for: Multiple clades of Husavirus in South America revealed by next generation sequencing
Source: PLoS One. 2021 Mar 23;16(3):e0248486. doi: 10.1371/journal.pone.0248486 (PMC7987173; doi:10.1371/journal.pone.0248486)
Supplement: Attachment — Submitted filename: PONE-responses.doc [file pone.0248486.s002.doc]

Dear Dr.
Gualtiero Alvisi, PhD
Academic Editor
PLOS ONE

We are happy with the comments made by the reviewers on the results presented in our manuscript ”Multiple clades of Husavirus in South America revealed by next generation sequencing of fecal samples (PONE-D-20-22053)”. We feel this paper is greatly improved after making the modifications as suggested by the reviewers. All sequences included in this study are available in the GenBank.

We also would like to thank the efforts of the editorial board for processing our manuscript.

Sincerely yours

Élcio Leal, MV, MSc, PhD

Institute of Life Sciences, Federal University of Pará;

Avenida Augusto Corrêa, 1;

CEP 66075-110;

Belém, PA, Brazil;

mobile/Phone-Fax: (55 91) 992677839 / 32017601;

Email: elcioleal@gmail.com, elcioleal@ufpa.br

Reviewers' comments:

Reviewer's Responses to Questions

**Comments to the Author**

Reviewer #1: The authors describe the identification and phylogenetic analysis of husaviruses for the first time in Brazil. There are some points that need to be clarified.

Lines 37-39 “Detailed molecular characterization indicated that the nucleotide divergence of Brazilian HuVs is less than 11%. On the other hand, the genetic distance between Brazilian sequences and the closest HuV previously described is 18%.”: In what genome region?
Resp: to calculate the genetic distance we have used the polyprotein region. This information has been included in the manuscript (line 256, We have used the polyprotein region of these genomes to calculated the genetic divergence of sequences in the clade A1 which is 18%.)
Line 104 “New Generation Sequencing (NGS)”: should be “Next Generation Sequencing”.
Resp: we have changed New to Next (line 128)
Line 177 “From the 251 samples analyzed by serology”: How did the authors analyzed by serology? This should be included in the Materials and Methods section.
Resp: We didn't perform the serological test in these individuals because only fecal samples were available. We did bacterial culture techniques and conventional parasitological tests besides PCR and NGS. This was corrected in the new version of the manuscript (118-128)
Table 1: Collection date should be included. “Parvovirus e”,” Rotavirus e”, ” Torque Teno Virus e”, and ” Adenovirus e” are correct?
Resp: We have included collection dates in table 1 and removed the typos “e”
Lines 193-195 “We found that the similarity of Brazilian sequences is 92% (in this calculation we excluded sequence TO-029 because it is incomplete). The highest similarity of a Brazilian sequence and the previously described husavirus was 87%,”: In what genome region?
Resp: To calculate the nucleotide similarity we have used the polyprotein regions of Husaviruses. We have changed the text accordingly. (line 219: We found that the nucleotide similarity in the polyprotein region of Brazilian sequences is 92%.)
Lines 242-244 “husavirus was initially detected in stool samples in the Netherlands and since then it has been reported in a low frequency in other countries such as Venezuela and Viet Nan [1,2,3,4].”: This sentence is a duplication of Introduction section lines 49-53.
Resp: In the new version of the manuscript, we have changed the text of the introduction section.
Lines 42-44 “The lack of geographical structure besides the occurrence of distinct lineages in the same region indicates that HuV is an old virus.”, lines 264-266 “More interesting is the presence of a very divergent clade of HuV (clade A2 in the tree) in South American, thus suggesting that husavirus was circulating in the native population before American colonization.”, lines 268-270 “The presence of multiple variants (clades) of husavirus in South America indicate an intricate evolutionary history perhaps with recurrent infection events between native people and colonizers.”: How did the authors estimate that husavirus was circulating in the native population? How did the authors eliminate the possibility of the viruses introduced from other countries or areas?
Resp: We have changed this sentence in the new version of the manuscript,
Figure 2: AB010145_Aichi_virus_genomic may be miss assigned.

Resp:The sequence with the genbank ID AB010145 is indeed a Aichivirus ( isolate A846/88)

Reviewer #2: The authors studied the molecular characterization of husavirus in faecal samples from patients with gastroenteritis in South America. They detected husavirus in only five samples. The authors showed that the viral strains are closely related based on their nucleotide sequence and are closely related to a HuV identified in Viet Nam in 2013. In general, the manuscript sounds good. Although there is nothing novel about the idea nor the techniques which were used to study the molecular characterization of the virus, it is a good study as it is the first on this virus in Brazil. The data of this study may add to global knowledge about the molecular epidemiology of Husavirus. There are crucial points that the authors should elucidate.

Resp: We have updated the sequences used for the comparative phylogenetic analysis and changed the manuscript accordingly.

**Review**

**PLOS One Journal**

**Multiple clades of Husavirus in South America revealed by next-generation sequencing of faecal samples**

The authors studied the molecular characterization of husavirus in faecal samples from patients with gastroenteritis in South America. They detected husavirus in only five samples. The authors showed that the viral strains are closely related based on their nucleotide sequence and are closely related to a HuV identified in Viet Nam in 2013. In general, the manuscript sounds good. Although there is nothing novel about the idea nor the techniques which were used to study the molecular characterization of the virus, it is a good study as it is the first on this virus in Brazil. The data of this study may add to global knowledge about the molecular epidemiology of Husavirus. There are crucial points that the authors should elucidate.

**Introduction:**

The authors should restructure the introduction as it lacks detailed information in addition to poor English.

1How was husavirus discovered in 2015 from human faecal samples? What were the techniques used?Resp: Posa-like viruses, including husavirus, were all identified mostly by NGS from faecal/environmental samples. The introduction has been updated accordingly.
2The authors stated that the virus could cause a variety of clinical conditions in the host, which host precisely? Human or non-human? Need more resources and references. Resp: We say that members of picornavirales order infect and cause diseases in a variety of hosts. Posa-like viruses, on the other hand, have been detected in faecal and environmental samples linked with animal hosts. However, there is no clear evidence Posa-like viruses cause disease or infect animals.
3What is the importance of studying the phylogenetic characterization of the virus? Resp: There are many important features we can obtain by phylogenetic studies of viruses. We can understand and elucidate relatedness and evolutionary aspects of viruses by inferring trees and performing measurements of branch lengths and other key parameters provided by evolutionary models. In our case, we have shown that in South America there are two divergent phyloclades (husavirus A1 and A2).
4More references should be included in the introduction related to the clinical importance of the virus. Resp: We have modified several sections of the manuscript and have added new references.

**Study population and specimen collection:**

- The statement `the protocol used failed to identify possible outbreak` needs more clarification.

Resp: The updated edition of the manuscript omitted this phrase because the experimental design was not aimed at exploring viral outbreaks.

**Sample screening:**

- It is recommended to screen the stool samples first for the presence of common viruses causing gastroenteritis by a routine diagnostic test such as multiplex-Real Time PCR and then perform NGS to identify the known or unknown viruses in the samples. Resp: Samples were screened for viral enteric pathogens (i.e., adenovirus, rotavirus and norovirus), using commercial enzyme immunoassays, such as RotaScreenII ®and AdenoScreen®EIA (Microgen Bioproducts Ltd, 1, Watchmoor Point, Watchmoor Rd, Camberley GU15 3AD, UK).

**Results:**

1The authors mentioned that 251 samples were analyzed by serology or NGS; however, they did not mention that they used serology in the methodology sectiond. Resp:Serology is these individuals was not done. Faecal samples were tested prior to NGS to assess the existence of viruses typically associated with diarrhoea using a commercial immunoassay enzyme. In order to make this detail understandable, we updated the manuscript.
1The authors did not remark what is the percentage of the identified HuV in the stool samples?Resp: In 5 samples out of 251 (5/251=1.99 percent) by PCR and NGS, we found Husavirus, which is in the abstract of the new version of the manuscript.
2Different viruses were detected in the clinical samples using NGS. Were they found as single viruses or co-infection?Resp: Table 1 summarizes the viral species found in individuals whom husavirus was detected.
3‘The highest similarity of the Brazilian sequences and previously described husavirus was 87%.......’. This statement should be restructured to be more understandable.Resp:4. In order to make clear that the similarity between TO-030 and KX673248 is 87 percent, we changed this sentence.
4It should be noted to the authors that all identified husaviruses from stool samples belong to clade A1 only, while clade A2 consists of reference husavirus A strains. Resp: Clade A2 sequences were obtained from Amerindians and in an Ethiopian child.
5The authors have just included seven human husavruses to perform the phylogenetic analysis. Where are the remaining 10 human HuV nucleotides sequences that are deposited in the GeneBank database? Including all the human 17 HuV nucleotides sequences will give a better image on the relatedness of the detected strains in this study to the other strains detected in other countries .Resp: We included 31 Husavirus sequences in this version of the manuscript and this gave us more information about the relatedness of this virus.

**Discussion:**

1The authors stated ‘the presence of a very divergent clade of HuV (clade A2) in South America, which suggest that husavirus was circulating in the native population’. This statement needs lots of explanations and confirmations. What are the other molecular analysis data that support this conclusion?Resp: In order to make it clear that husavirus is an ancient virus, we have changed the discussion and there is no clear indication that it was in native Amerindians before colonization.
2Since the HuV strains detected in this study were found as co-infection with other viruses causing gastroenteritis, how can the authors confirm that HuV able to cause clinical outcome as a sole virus? It may be dependant virus! Resp: We didnt say husavirus is causing gastroenteritis we mention that rotavirus is the likely the pathogen causing disease in these children (lines 298-300).
3The authors did not discuss the limitations of this study. Resp: There is no case-control trial, aside from the small number of samples and skewed samples that involve only children with gastroenteritis. It is also important to remember that our research was not designed to establish whether husavirus infects human cells, while we demonstrate that helminths are not the source of husaviruses.
