# Supplementary material for: Multiple clades of Husavirus in South America revealed by next generation sequencing
Source: PLoS One. 2021 Mar 23;16(3):e0248486. doi: 10.1371/journal.pone.0248486 (PMC7987173; doi:10.1371/journal.pone.0248486)
Supplement: Attachment — Submitted filename: Response to Reviewers.doc [file pone.0248486.s004.doc]

Dear Gualtiero Alvisi
Academic Editor

PLOS ONE

We are glad to know that our manuscript “**Multiple clades of Husavirus in South America revealed by next generation sequencing**” has been considered within the scope of the PLOS ONE.

We deeply appreciated the detailed and insightful comments made by the referee. We also would like to thank the efforts of the editorial board for processing our manuscript. All efforts were made to enhance the quality of the manuscript and to allow for its expedient publication. We are sure that this paper is greatly improved after making the modifications suggested by the reviewer.

Sincerely

Elcio Leal

Editor Comments:

**Line 33. To recognize unknown causes of viruses – please rephrase.**

Resp: We have done the change this paragraph.

**Line 35. Using culture methods and parasitological tests to classify other enteric pathogens such as bacteria, parasites, and viruses, all samples were also examined. – please change to “****all samples were also analyzed using culture methods and parasitological tests to classify other enteric pathogens such as bacteria, parasites, and viruses”.**

Resp: We have done the change this paragraph.

Line 37. In 1.9 % of the samples, we observed HuV in three males and two females with a mean age of 2 year. – please change to “1.9 % of the samples samples tested positive for HuV, for a total of 5 positive children, with with a mean age of 2 year, with three males and two females.”

Resp: We have done the change this paragraph.

Line 39. The genetic gap between Brazilian sequences and the closest HuV mentioned previously, on the other hand, is 18 %. Please change to “The genetic gap between Brazilian sequences and the closest HuV reported previously, on the other hand, is 18 %.”

Resp: We have done the change this paragraph.

Furhermore Figures 1 and 2 are appear to have been inverted.

Resp: We have change this and images now correspond to the figure 1 and figure 2.

Reviewer 1

Lines 114-117 “Samples were screened for viral enteric pathogens (i.e., rotavirus and norovirus), using commercial enzyme immunoassays, such as RotaScreenII® and AdenoScreen®EIA (Microgen Bioproducts Ltd, 1, Watchmoor Point, Watchmoor Rd, Camberley GU15 3AD, UK).”: The authors mentioned “Samples were screened for viral enteric pathogens (i.e., rotavirus and norovirus), using commercial enzyme immunoassays”; however, they used “RotaScreenII® and AdenoScreen®EIA”. How did they screen for “norovirus”?

Resp: We have investigated norovirus by NGS and PCR. Results of this screening were published recently (Arch Virol. 2021 Mar;166(3):905-913. doi: 10.1007/s00705-020-04944-5. Epub 2021 Jan 19.PMID: 33462673).

Line 122 and elsewhere “Husavirus” or “husavirus”: “husavirus” (lines 230, 232, 235, 236, 246, 261, 265, 266, 267, 269, 273, 291, 292, 300, 305, 307, 308, 310, 315, 323, 326, 327, 328, 329, 330, and 331) should be “HuV”.

Resp: We have changed husavirus to HuV.
